# Normocaloric Diet Restores Weight Gain and Insulin Sensitivity in Obese Mice

**DOI:** 10.3389/fendo.2016.00049

**Published:** 2016-05-27

**Authors:** Giovanni Enrico Lombardo, Biagio Arcidiacono, Roberta Francesca De Rose, Saverio Massimo Lepore, Nicola Costa, Tiziana Montalcini, Antonio Brunetti, Diego Russo, Giovambattista De Sarro, Marilena Celano

**Affiliations:** ^1^Department of Health Sciences, University “Magna Graecia” of Catanzaro, Catanzaro, Italy; ^2^Department of Medical and Surgical Sciences, University “Magna Graecia” of Catanzaro, Catanzaro, Italy

**Keywords:** insulin resistance, obesity, Glut4, diet, glucose

## Abstract

An increased incidence of obesity is registered worldwide, and its association with insulin resistance and type 2 diabetes is closely related with increased morbidity and mortality for cardiovascular diseases. A major clinical problem in the management of obesity is the non-adherence or low adherence of patients to a hypocaloric dietetic restriction. In this study, we evaluated in obese mice the effects of shifting from high-calorie foods to normal diet on insulin sensitivity. Male C57BL/6JOlaHsd mice (*n* = 20) were fed with high fat diet (HFD) for a 24-week period. Afterward, body weight, energy, and food intake were measured in all animals, together with parameters of insulin sensitivity by homeostatic model assessment of insulin resistance and plasma glucose levels in response to insulin administration. Moreover, in half of these mice, *Glut4* mRNA levels were measured in muscle at the end of the high fat treatment, whereas the rest of the animals (*n* = 10) were shifted to normocaloric diet (NCD) for 10 weeks, after which the same analyses were carried out. A significant reduction of body weight was found after the transition from high to normal fat diet, and this decrease correlated well with an improvement in insulin sensitivity. In fact, we found a reduction in serum insulin levels and the recovery of insulin responsiveness in terms of glucose disposal measured by insulin tolerance test and *Glut4* mRNA and protein expression. These results indicate that obesity-related insulin resistance may be rescued by shifting from HFD to NCD.

## Introduction

Modern lifestyle is often characterized by sedentary activities and overeating. As a consequence, in the last decades, this has been responsible for the increased incidence and prevalence of obesity and obesity-induced comorbidities, such as insulin resistance and metabolic syndrome (1, 2) that may contribute to type 2 diabetes mellitus (T2DM) and cardiovascular disease (3). Several studies have demonstrated that a healthy lifestyle can lead to weight loss and improve insulin sensitivity (4–7). In this regard, a crucial role is played by the nutrient composition of the diet, both in terms of total caloric intake and the variety of its components, with particular attention to the different types of fatty acids (8, 9). Unfortunately, most of anti-obesity interventions are often limited by the difficulty to maintain a low-calorie dietary regimen, especially when long-term treatments are required (10, 11). Thus, few anti-obesity programs have been found to be helpful.

To date, several animal models have been used to evaluate the effects of various dietetic regimens on body weight and metabolic parameters. A validated experimental model is represented by mice fed with a high fat diet (HFD), which develop obesity, insulin resistance, and dyslipidemia (8–13).

In the present study, we evaluated the effects of the transition from HFD to normocaloric diet (NCD) (regular food with no additive agents or nutraceutical compounds) on body weight and insulin responsiveness in C57BL/6JOlaHsd mice, a strain of mice genetically prone to develop obesity and insulin resistance (14).

## Materials and Methods

### Animals and Study Design

**Figure 1:**
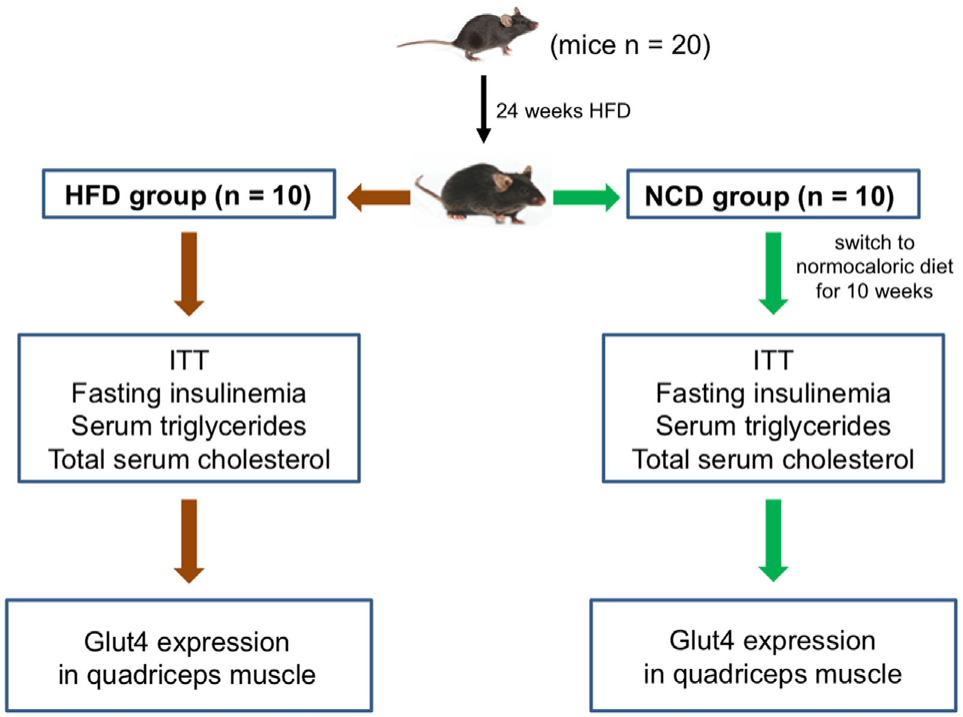
**Study design**. A schematic representation of the study protocol and experimental plan is shown.

Five-week-old male C57BL/6JOlaHsd mice (*n* = 20), NCD and HFD, were purchased from Harlan Laboratories S.r.l (Udine, Italy). Mice were housed in individual cages and maintained on 12-h light/dark cycle at 21 ± 1°C and 50 ± 5% humidity with free access to water and food *ad libitum*. Animals were fed with HFD containing 60.3% kcal fat, 21.3% kcal carbohydrate, and 18.4% kcal protein (HFD group) for 24 weeks. After this period, 10 mice were euthanized by cervical dislocation and the other 10 were fed with NCD only (Teklad Global 18% kcal fat, 58% kcal carbohydrate, and 24% kcal protein) (NCD group) for the subsequent 10 weeks. A schematic representation of the study design is shown in Figure 1. Body weight, girth waist, and food intake were recorded at weekly interval for all animals (15). Liver, skeletal muscle, and abdominal fat were excised, weighted, and stored in liquid nitrogen. This study was performed following the Italian (D.M. 116/92) and ECC regulations (O.J. of E.C.L 358/1 12/18/1986), in accordance with the guide for the care and use of laboratory animals and approved by the local ethical committee.

### Biochemical Analysis

Blood samples were collected after 12 h of fasting. Serum was separated by centrifugation at 1700 *g* for 10 min at room temperature and stored at −20°C, until use. Total cholesterol and triglycerides were measured using commercial reagents (Siemens Healthcare Diagnostics, Milano, Italy) and an automated biochemistry analyzer (Dimension EXL, Siemens Healthcare Diagnostics). Insulin levels were measured using ELISA kit (Rat/Mouse Insulin ELISA Kit, EMD Millipore Corporation, Darmstadt, Germany), according to the manufacturers’ instructions.

### Insulin Tolerance Test

Insulin tolerance test (ITT) was performed in both HFD and NCD groups, as previously described (16). Animals were fasted for 12 h, weighed, and injected intraperitoneally with insulin (1 U/kg body weight Regular^®^, Novorapid, Novonordisk, Roma, Italy). Blood glucose levels were measured after 0, 15, 30, 60, and 90 min using an automatic glucometer (Glucocard, Menarini Diagnostics, Firenze, Italy).

### Expression of Glucose Transporter Type 4

Total RNA was isolated from quadriceps skeletal muscle using TRIzol reagent (Life Technologies, Monza, Italy), following the manufacturer’s recommended protocol and quantified with a NanoDrop Spectrophotometer (Thermo Fisher Scientific, Inc., Waltham, MA, USA). RNA levels were normalized against 18S ribosomal RNA in each sample, and cDNAs were synthesized from 1 μg of total RNA using the High Capacity cDNA Reverse Transcription Kit (Life Technologies). Primers for mouse *Glut4* and *ribosomal protein S9* (*RPS9*) were designed according to sequences from the GenBank database. Relative quantification was made using a real-time thermocycler (Eppendorf Mastercycler ep realplex, Milano, Italy). In a 20-μl final volume, 1 μl of cDNA solution was mixed with SYBR Green RealMasterMix (Eppendorf) and 0.2 μM of each sense and antisense primers. SYBR Green fluorescence was measured, and relative quantification was made against either RPS9 or *Gapdh* cDNAs, used as internal standards. All PCR reactions were carried out in triplicates. Glut4 protein expression was measured in quadriceps muscle from six to eight mice of each group, using a rabbit anti-Glut4 polyclonal antibody as previously described (17).

### Statistical Analysis

Results are expressed as mean ± SD. The independent *t*-test was used to evaluate intergroup differences. All statistical analyses were performed using GraphPad Prism version 5.0 statistical software (GraphPad Software Inc., San Diego, CA, USA). *p* values lower than 0.05 were considered statistically significant.

## Results

### Effects of Normocaloric Diet on Body Weight and Biochemical Parameters

**Figure 2:**
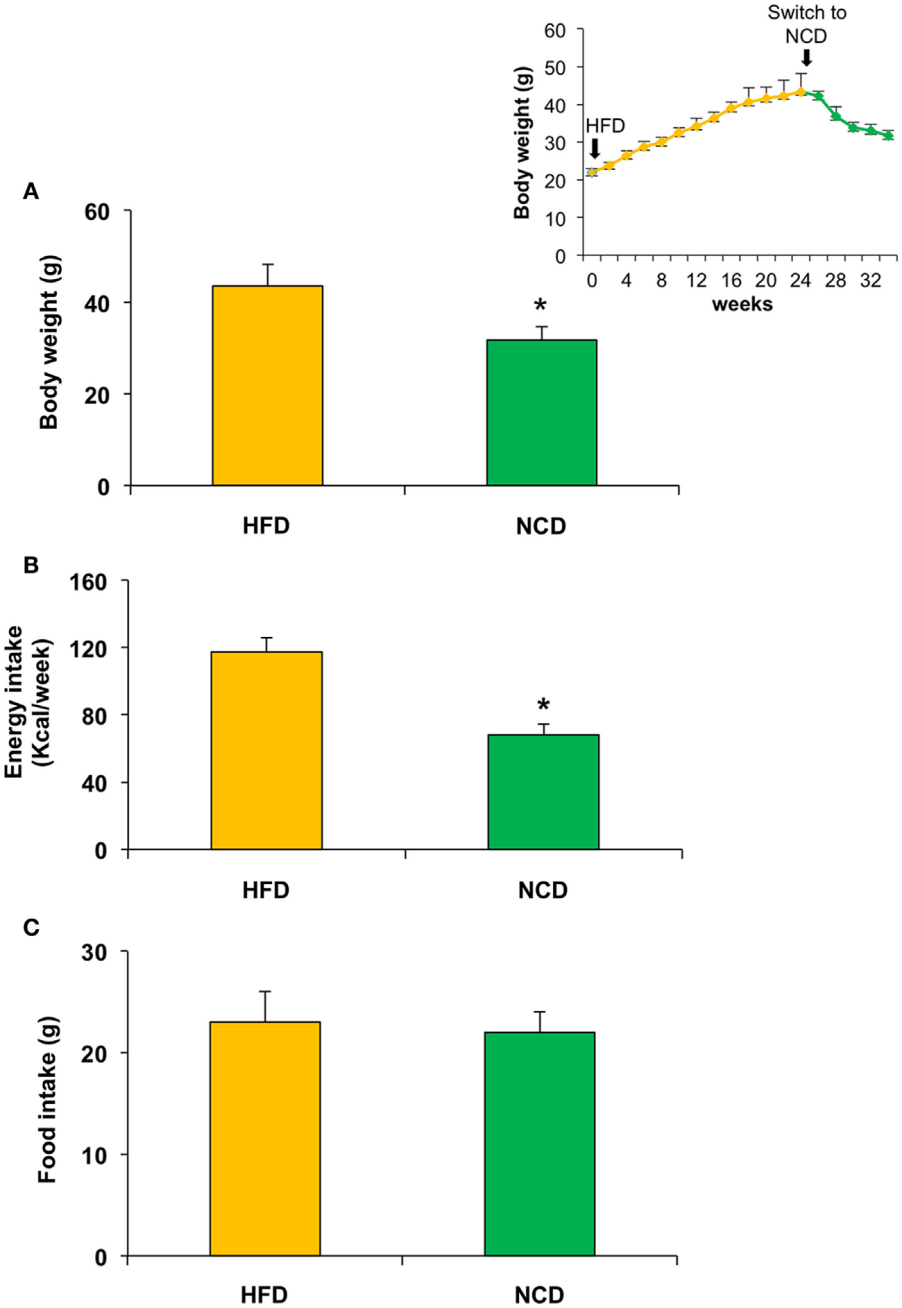
**Effects on body weight, food, and energy intake in mice fed with high fat diet (HFD) for 24 weeks and with normocaloric diet (NCD) for other 10 weeks**. A significant reduction of body weight and energy intake was observed in NCD mice **(A,B)**, whereas no significant difference was detected in food intake **(C)**. Body weight over the time is shown in the inset. Values are expressed as mean ± SD. *p < 0.001.

**Figure 3:**
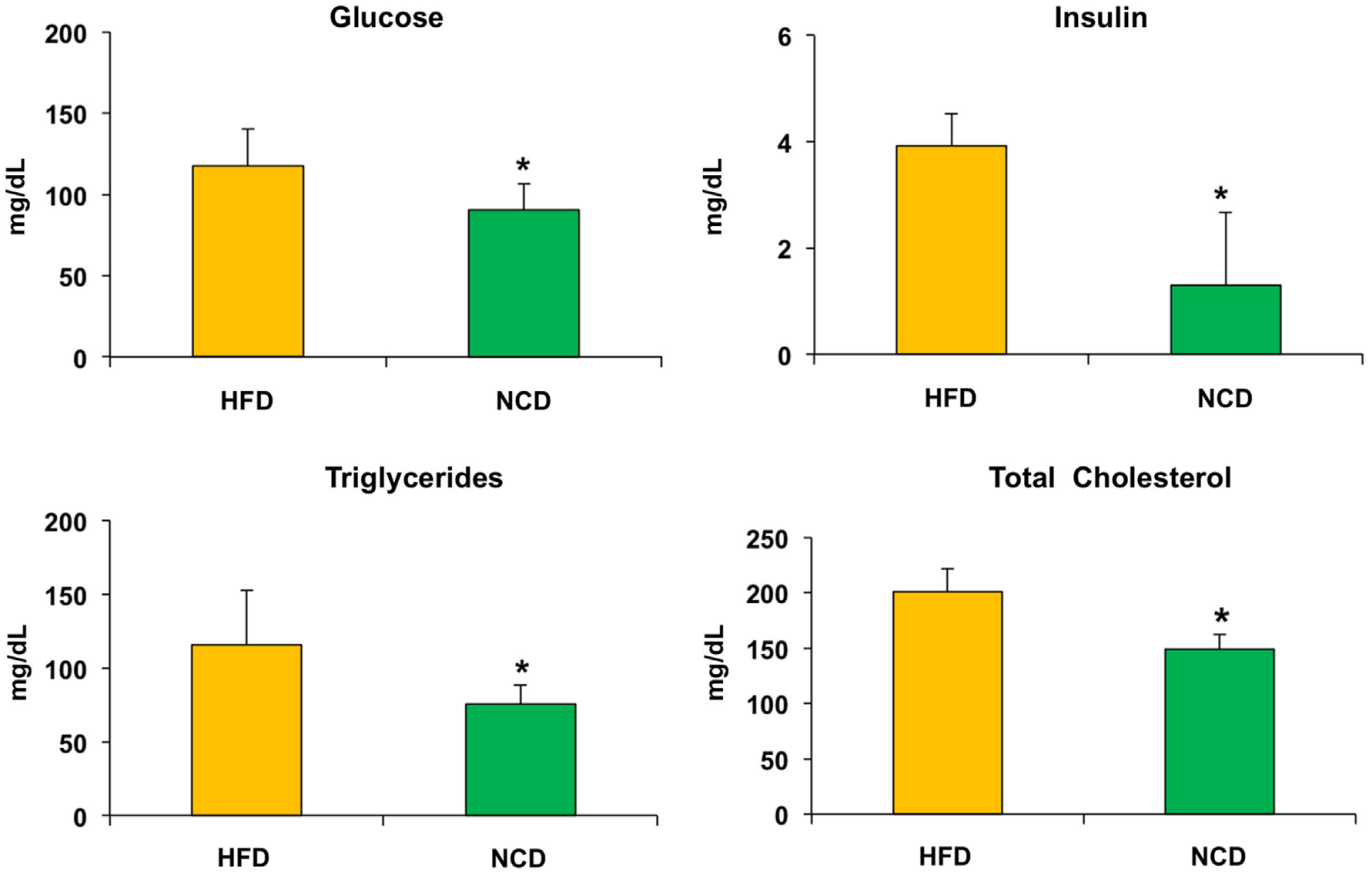
**Biochemical parameters**. Blood samples were collected as indicated in Section “Materials and Methods.” After 10 weeks of feeding with a normocaloric diet (NCD), mice showed a significant reduction of plasma glucose levels and serum insulin levels, as well as a reduction in both triglycerides and total cholesterol when compared to the HFD. Values are expressed as mean ± SD. *p < 0.05.

Twenty mice were fed with HFD (HFD group) for 24 weeks, reaching a weight of approximately 43 g, with fasting plasma glucose levels between 90.5 and 117.7 mg/dL, which were consistent with a condition of impaired fasting glucose. After the 24-week period, half of the mice were fed with NCD for the following 10 weeks (NCD group). A significant decrease of body weight was observed in the NCD group compared to the HFD group (27%, *p* < 0.001), as a result of the decrease in energy intake due to the less caloric supply derived from the NCD rather than the different food intake (Figure 2). In the NCD group, we also observed a decrease in liver size, fat depots, and girth waist (Table 1). Moreover, shifting to NCD resulted in a significant decrease in plasma glucose levels (*p* < 0.05) and serum insulin levels (*p* < 0.01), as well as triglycerides (*p* < 0.05) and total cholesterol (*p* < 0.05) (Figure 3).

**Table 1:** **Weight and waist in HFD and NCD mice**.

	HFD	NCD	*p* value
Liver (g)	1.24 ± 1.09	1.09 ± 0.09	<0.05
White adipose (g)	1.54 ± 0.31	1.29 ± 0.08	<0.05
Epididymis (g)	0.60 ± 0.16	0.33 ± 0.08	<0.01
Girth waist (cm)	10.55 ± 0.42	9.54 ± 0.17	<0.01

*Values are expressed as mean ± SD*.

### Effects on Insulin Sensitivity

**Figure 4:**
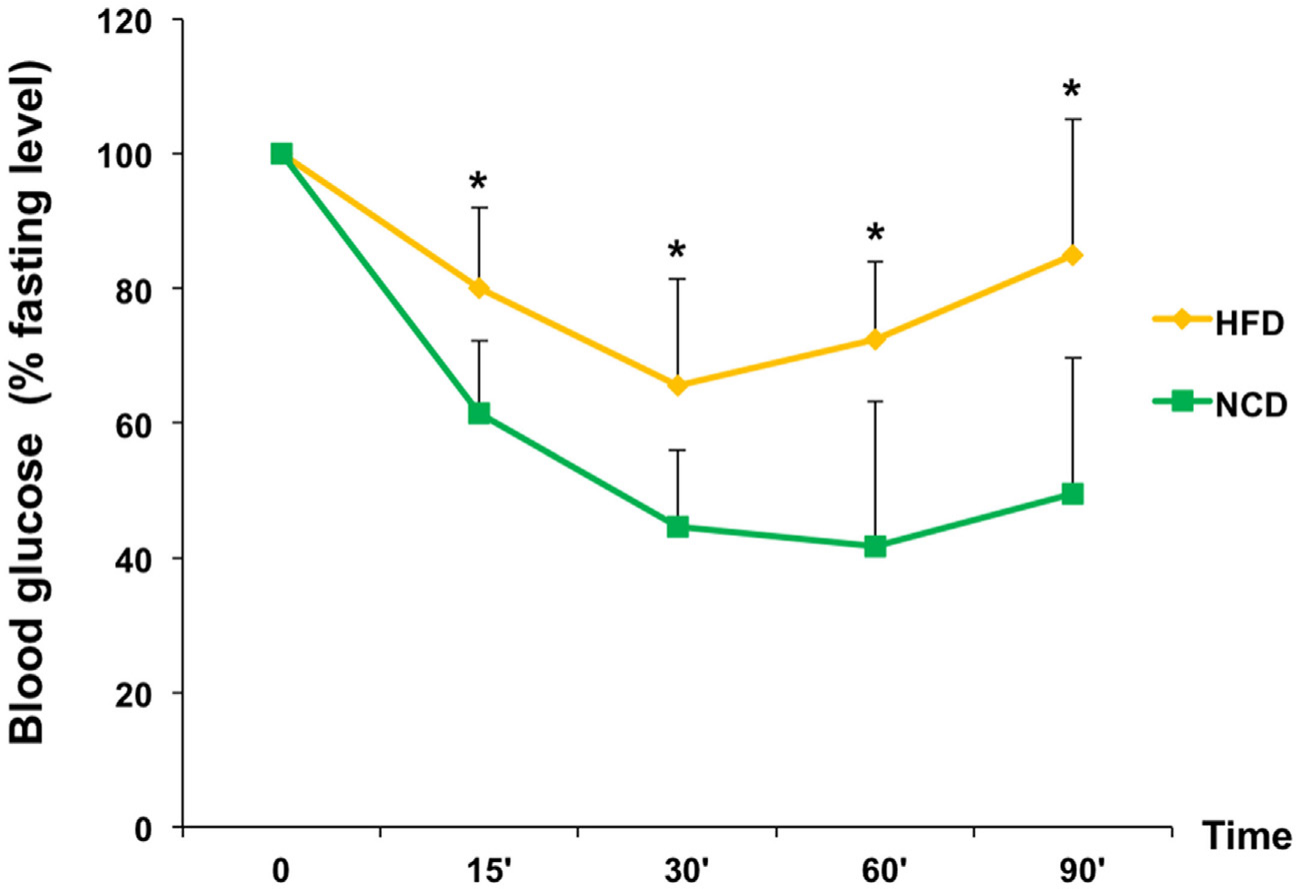
**Insulin sensitivity**. HFD and NCD mice fasted for 12 h were injected intraperitoneally with insulin (1 U/kg). Blood glucose levels were measured with a glucometer, as reported in Section “Materials and Methods.” Values are expressed as mean ± SD. *p < 0.05.

**Figure 5:**
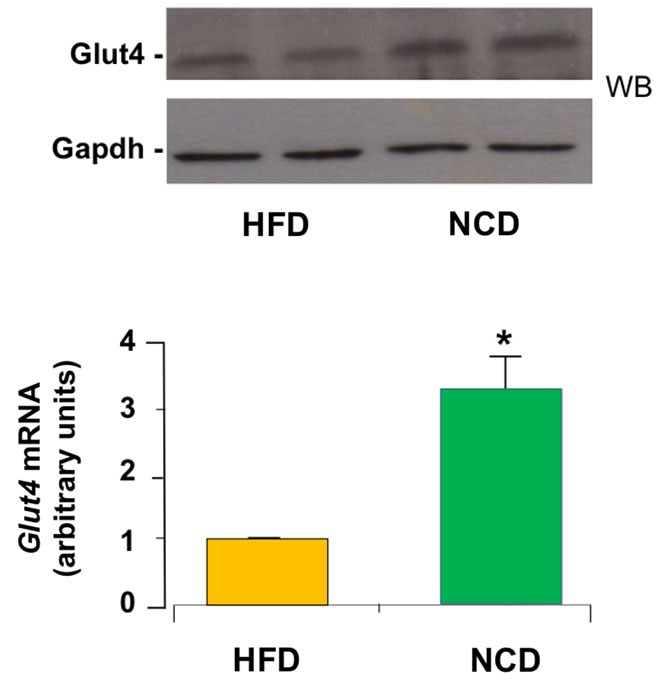
**Expression of Glut4**. Glut4 mRNA levels were measured by qRT-PCR in skeletal muscle from HFD and NCD mice, after insulin stimulation. Results are the mean ± SD for six animals per group. *p < 0.05 versus HFD mice. A representative Western blot (WB) of Glut4 in quadriceps muscle from six to eight mice of each group is shown in duplicate in the autoradiogram. Gapdh, control of protein loading.

Next, we evaluated the effects of NCD on insulin sensitivity. ITT performed in mice before and after NCD showed a better response to insulin in terms of changes in blood glucose concentrations in the NCD group than in the HFD group. In fact, the glucose-lowering effect of exogenous insulin was enhanced in NCD mice during ITT and was reduced in HFD mice (Figure 4). From a mechanistic point of view, the improvement in insulin sensitivity in mice in response to NCD was dependent, at least in part, on an increase in *Glut4* expression induced in skeletal muscle following the transition from HFD to NCD. To show such a molecular link between restoration of insulin sensitivity and NCD, total RNA was extracted from skeletal muscle of animals before and after shifting to NCD, after insulin stimulation, and *Glut4* mRNA and protein levels were measured. As shown in Figure 5, both insulin-stimulated Glut4 mRNA and protein expression were significantly increased in skeletal muscle of NCD mice as compared with that of HFD mice (*p* < 0.05).

## Discussion

Obesity is a chronic disorder that can cause other health problems, such as diabetes, hypertension, hepatic steatosis, obstructive sleep apnea, and atherosclerosis (18). The association of obesity with T2DM is well established, due to the negative influence of excessive body fat on peripheral insulin action and hepatic function, leading to insulin resistance (19). Treatment of obesity includes hypocaloric diet, exercise, and lifestyle modifications, with dietary manipulation still representing the first-line therapeutic approach for this common disorder (20, 21). However, it is still debated which is the more appropriated dietetic regimen to obtain a weight loss, which may be at the same time rapid, well tolerated, and sustainable for a long period of time. Although the importance of calorie restriction in this condition is well recognized, also for the positive psychological benefit for the patient and the family, there is no doubt that a major problem in treating obesity is still represented by the relatively low level of adherence of affected subjects to low/very low-calorie diets (22–24). Thus, many dietary strategies have been proposed to overcome such obstacles, but the results are not satisfactory enough in most of obese patients (25, 26). In these individuals, we hypothesized that shifting to normocaloric balanced diet, formulated to avoid excess fat, rather than hypocaloric diet – which would obtain a better compliance especially in view of long-term treatment – might be sufficient, in addition to physical exercise and lifestyle change, to get more satisfactory results in terms of weight loss and consequent improvement in obesity-related insulin resistance. This hypothesis is well supported by the present finding in our mouse model of obesity and obesity-induced insulin resistance. In fact, shifting from HFD to NCD for 10 weeks, caused a significant reduction of body weight mainly due to the reduction of visceral fat, together with the overall reduction of triglycerides, total cholesterol, and, most importantly, restoration of insulin sensitivity, as reflected by the decline in fasting insulin levels. A similar approach treating obese mice with NCD has also been used in a few other studies where, however, some nutraceutical compounds or other ingredients were added to regular food (27–30). This is slightly different than what we did in our study, in which NCD itself, without any additive agent, was able to improve insulin sensitivity and Glut4 expression.

*Glut4* is the major insulin-dependent glucose transporter in muscle. Abnormalities at this level are a hallmark of peripheral insulin resistance (31). In the present study, the improvement in insulin sensitivity associated with increased *Glut4* mRNA expression in NCD mice provides a possible mechanistic explanation as to how the normal calorie diet can improve insulin responsiveness and supports the hypothesis that rescue from insulin resistance and diabetes can be reached without the adoption of a low-calorie diet. If confirmed in obese humans, such an approach, in association with adequate and individualized physical exercise programs, might be able to contribute to counteract the long-term failure of the current therapeutic approaches adopted in these individuals, and this would confirm further the appropriateness of mouse models for studying human obesity. However, on the other hand, it is also known that marked interspecies differences exist between human and mouse with respect to behavioral control of food uptake, tissue energy disposal and storage, weight, and weight loss, which emphasize the influence of non-genetic environmental factors and genetic modifiers in determining the phenotypic variations observed in humans and animal models of obesity. Thus, caution is required in generalizing these findings. As a limitation of the present work, the fact is that mice of different ages were compared in our study.

In conclusion, numerous anti-obesity initiatives have been adopted up to now, which include lifestyle changes, drug treatments, and surgery. However, because of the limited efficacy and the occurrence of adverse events in affected treated patients, alternative and complementary therapies for weight loss have been investigated, including acupuncture, dietary supplements, etc. Our findings in the current work provide valuable information about the efficacy of shifting to NCD in restoring weight and insulin sensitivity in HFD-induced obese mice. Similar studies in obese humans would reveal whether this strategy, probably better accepted by patients, may be successful in correcting weight gain and obesity-related insulin resistance.

## Author Contributions

GEL contributed to animal testing and drafting of the manuscript; RFDR elaborated figures and tables and contributed to the analysis of the results; SML contributed to animal testing and drafting of the manuscript; BA performed the molecular analysis; NC performed the operation on the animals and supervised the animals’ maintenance during the treatment period; TM and GDS reviewed the final version of the manuscript; AB contributed to the conception of the idea and critically reviewed the manuscript; DR contributed to the conception of the idea, drafted the manuscript, and critically reviewed the final manuscript; and MC contributed to animal testing, analysis of the results, and editing of the manuscript. All authors read and approved the submitted version.

## Conflict of Interest Statement

The authors declare that the research was conducted in the absence of any commercial or financial relationships that could be construed as a potential conflict of interest.
